# Interaction between Flavonoids and Carotenoids on Ameliorating Oxidative Stress and Cellular Uptake in Different Cells

**DOI:** 10.3390/foods10123096

**Published:** 2021-12-14

**Authors:** Xuan Chen, Zeyuan Deng, Liufeng Zheng, Bing Zhang, Ting Luo, Hongyan Li

**Affiliations:** 1 State Key Laboratory of Food Science and Technology, University of Nanchang, Nanchang 330047, China; 352335918030@email.ncu.edu.cn (X.C.); dengzy@ncu.edu.cn (Z.D.); zhenglf2018@ncu.edu.cn (L.Z.); zhangbingair@ncu.edu.cn (B.Z.); ting.luo@ncu.edu.cn (T.L.); 2 Institute for Advanced Study, University of Nanchang, Nanchang 330031, China

**Keywords:** antioxidant, synergism, cell uptake, flavonoids, carotenoids

## Abstract

Flavonoids (quercetin, luteolin) and carotenoids (lycopene, lutein) were combined at different molecular ratios in a total concentration of 8 μM to investigate their antioxidant interactions. Cellular uptake of carotenoids, the expression of carotenoid transporters, the ROS scavenging ability, and antioxidant enzymes activities were compared in HUVEC, Caco-2, and L-02 cells. Combinations with flavonoids in the majority showed stronger antioxidant activity. Lycopene combined with quercetin at ratio 1:5 showed stronger ROS scavenging activities, increased 18, 12, and 12 Cellular antioxidant activity (CAA) units in HUVEC, Caco-2, and L-02 cells, respectively, and promoted SOD and CAT activities than individual component. The cell uptake of carotenoids was enhanced by flavonoids in antioxidant synergistic groups, while dampened by flavonoids in antagonistic groups in HUVEC cells. The synergistic group (lycopene:quercetin = 1:5) increased lycopene uptake by 271%, while antagonistic group (lutein:quercetin = 5:1) decreased lutein uptake by 17%. Flavonoids modulated the effects of carotenoids on the expression of active transporters scavenger receptor class B type I (SR-BI) or Niemann-Pick C1-like 1 (NPC1L1). The synergistic group (lycopene:quercetin = 1:5) increased the expression of SR-BI compared to individual lycopene treatment in HUVEC and Caco-2 cells. Thus, a diet rich in both flavonoids and lycopene possesses a great antioxidant activity, especially if a higher amount of flavonoids is included.

## 1. Introduction

Epidemiology studies have suggested the benefit effects of intaking whole foods, such as fruits and vegetables, in preventing many chronic diseases, including diabetes, atherosclerosis, inflammatory bowel disease, and Alzheimer’s disease [1,2]. Phytochemicals, such as flavonoids and carotenoids, are identified as bioactive compounds in vegetables and fruits that may contribute to those health benefits [3]. However, evidence has accumulated that consuming food bioactive compounds such as phytochemicals cannot be comparable to the observed benefits of whole food diets rich in fruits, vegetables, or legumes [4]. It was postulated that phytochemicals and other bioactive compounds in whole food form complexes or work synergistically to exert the enhanced effects on antioxidant, anti-inflammatory, anti-carcinogenic activities, anti-diabetes, and neuroprotection [5]. The interactions (synergism, additivity, and antagonism) and the implication on bioactivities of components from foods have been studied in various aspects (chemical-based model, cell model, animal study, etc.) [6,7,8,9,10]. However, there is a gap between the interactions assessed by chemical-based models and biological-related models. It may be due to the different reaction environments, as the results collected by biological-related models often reflected the influence of bioavailability and bioaccessibility, metabolic environment, and cell signaling regulation on the interactions of bioactive compounds.

Bioavailability and bioaccessibility are critical to bioactivity, since the nutrients must be absorbed, distributed to different cells or tissues, and metabolized before they exert biofunctions. Evidence has accumulated that co-treatment with other carotenoids or other types of nutrients such as fat-soluble vitamin, could facilitate or interfere with the cell uptake of carotenoids [11]. It may further result in a change of bioactivities. Yet the link between the interactions among the co-exist compounds in whole foods on the absorption and their combined effects on oxidative prevention remains unclear. Few researchers related the absorption with bioactivities among co-exist compounds in natural foods. Phan et al. [12] revealed that the presence of anthocyanins hampered the antioxidant and anti-inflammatory effects of β-carotene, while anthocyanins increased the absorption of β-carotene by 68% to 200%. The increase of β-carotene uptake to a particular concentration might induce the pro-oxidant activity of β-carotene. This effect could be partly contributed to the antagonism in the combinations. Nonetheless, it is still unclear how phytochemicals influenced the absorption of co-exist plant nutrients and resulted in antioxidant synergy or antagonism. The influence of cell type is often neglected, which is indeed important as metabolic activity and cell signaling usually vary in different cell types.

**Figure 1:**
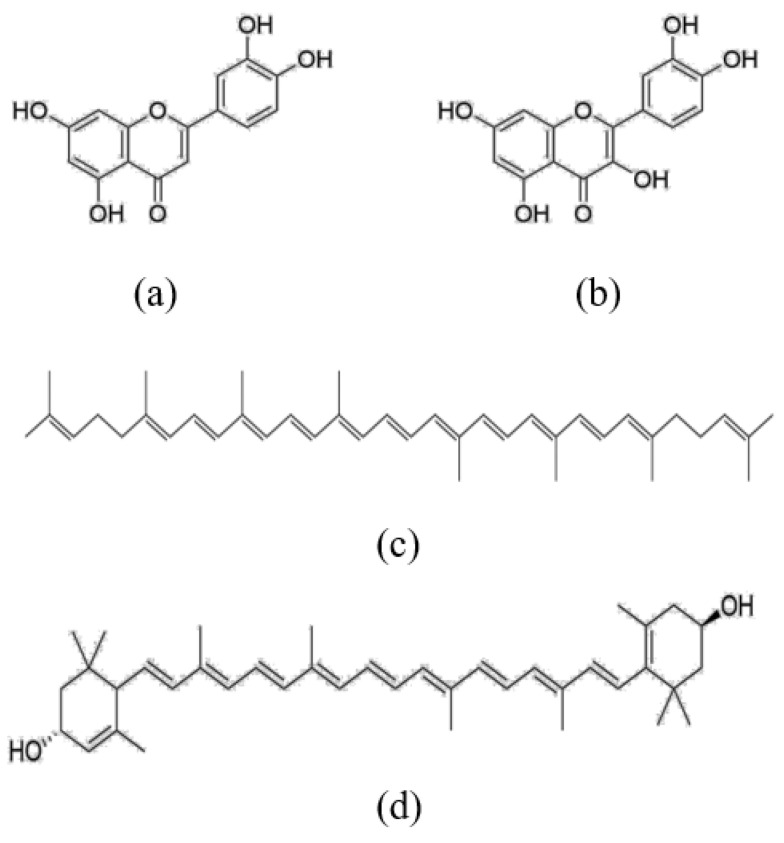
Structure of flavonoids and carotenoids used in this study. (**a**) quercetin, (**b**) luteolin, (**c**) lycopene, (**d**) lutein.

Quercetin and luteolin (Figure 1) are two most bioactive flavonoids and excellent antioxidants ubiquitously found in plant foods, such as apples, peppers, and citrus fruits. They have diverse biological functions, including antioxidant, anticancer, and anti-inflammatory properties [13]. They own multiple hydroxyl groups on their basic benzo-pyrone (C6-C3-C6) moiety, which render them strong antioxidant activity. Lycopene and lutein (Figure 1) are abundant in plasma, and they are rich in green leafy vegetables, corn, tomatoes, and watermelons [14]. They were also reported to inhibit inflammation, lower the risk of arthrosclerosis, ischemia/reperfusion injury, and neurodegenerative diseases through suppressing oxidative stress [15]. The potent antioxidant activity relies on their conjugated double-bond structure. Carotenoids are absorbed through simple diffusion across the membrane of epithelial cells or with the help of transporters, such as scavenger receptor class B type I (SR-BI) and Niemann-Pick C1-like 1 (NPC1L1) [16].

In this study, the antioxidant interactions of flavonoids (quercetin, luteolin) and carotenoids (lycopene and lutein) were studied in the human umbilical vein endothelial cells (HUVEC), human colon carcinoma cells (Caco-2), and the human fetal hepatocyte cells (L-02). Further, the influence of the presence of flavonoids on carotenoids uptake and on the expression of carotenoids transporter SR-BI and NPC1L1 were investigated to unveil the relationship between antioxidant interactions and the cell uptake of phytochemicals.

## 2. Materials and Methods

### 2.1. Materials and Reagents

2,2′-Azobis (2-amidinopropane) dihydrochloride (AAPH), Hydrogen peroxide (H_2_O_2_) solution (30 wt.% in H_2_O), quercetin, luteolin (purity ≥ 99%) were purchased from Shanghai Aladdin Reagents Co., (Shanghai, China), TritonX-100, lutein, lycopene (purity ≥ 99%), Hank’s balance salt solution was purchased from Solarbio technology Co., (Beijing, China). Quercetin and luteolin were dissolved in dimethyl sulfoxide (DMSO), lutein and lycopene were dissolved in tetrahydrofuran (THF, Damao Co., Ltd., Tianjing, China) at a concentration of 10 mM, respectively, and then freshly diluted in culture medium with 5% fetal bovine serum (FBS). The final concentration of DMSO and THF in culture medium was below 0.1% (*v*/*v*) and 0.05% (*v*/*v*), respectively. Dulbecco’s modified Eagle’s medium (DMEM), Roswell Park Memorial Institute (RPMI) 1640 medium, and FBS were obtained from Biological Industries, Shanghai, China. Ham’s F-12K (Kaighn’s) Medium (F-12K) was purchased from Procell Life Science and Technology Co., Ltd. (Wuhan, China). Anti-Niemann-Pick C1-like 1 (NPC1L1) ab124801 and Anti-Scavenging Receptor (SR-BI) antibody ab217318 were obtained from Abcam Ltd. (Cambridge, UK). Anti-β-actin HC201-01 was obtained from TransGen Biotech Co., (Beijing, China). Secondary antibodies horseradish peroxidase-conjugated anti-rabbit (L3012) or anti-mouse (L3032) were purchased from Signalway Antibody (Nanjing, China). 2′,7′-Dichlorodihydrofluorescein diacetate (DCFH-DA) were purchased from Sigma-Aldrich ((St. Louis, MO, USA). Super-enhanced chemiluminescence detection reagent was purchased from Beyotime Institute of Biotechnology (Shanghai, China). HUVEC, Caco-2, and L-02 cell lines were obtained from Procell Life Science and Technology Co., Ltd. (Wuhan, China).

### 2.2. Cell Culture and Treatments

HUVEC, Caco-2, and L-02 cells were selected to mimic the antioxidant interactions of flavonoids and carotenoids in cardiovascular system, small intestine, and liver, and they were cultured in F-12K, DMEM, and RPMI 1640 medium containing 10% (*v*/*v*) FBS, respectively. HUVEC and L-02 were cultured for 48 h before treatments, and Caco-2 were maintained for 5–7 days prior to various experiments. 1% penicillin/streptomycin were added in all mediums. Cells were incubated at 37 °C in a humidified incubator with 95% air and 5% CO_2_. The chosen concentration of quercetin, luteolin, lycopene, lutein was 8 μM according to their cytotoxicity (Figures S1–S3), and the concentration of H_2_O_2_ used to induce approximately 50% oxidative damage were 800 μM (L-02 cells), 1500 μM (HUVEC cells), and 2000 μM (Caco-2 cells) depending on the cell viabilities (Figure S4). 0.1% DMSO or THF were added in control groups in cell models.

### 2.3. Cell Viability Assay

Cell viability was determined by the CCK-8 assay, which is a cell proliferation assay using WST-8 cleavage. In brief, 100 μL of HUVEC or L-02 cells (1 × 10^5^ cells per well) was plated into a 96-well plate and reached 80–90% confluence before treatments, and 100 μL of Caco-2 cells was plated and reached 100% confluence before treatments. After pre-treated with or without different concentrations of quercetin, luteolin, lycopene, lutein at a series of concentrations (1–50 μM) for 12 h, cells in each well were then reacted with 10% CCK-8 for 1 h. Absorbance was then recorded at 450 nm using microplate reader (Thermo Scientific Varioskan Flash, Vantaa, Finland). Cell viability (%) = [A (sample) − A (background)]/[A (control) − A (background)] × 100.

### 2.4. Cellular Antioxidant Activity

The cellular antioxidant activity (CAA) assay was referred to the methods from Wolfe and Liu [14] with slight modification. HUVEC, Caco-2, and L-02 cells were seeded at a density of 1 × 10^5^ per well on a 96-well microplate. After reaching confluency, triplicate wells were treated with 100 μL a total concentration of 8 μM of quercetin, luteolin, lycopene, lutein, or their combinations and co-incubated with 30 μM DCFH-DA for 1 h and then replaced by 100 μL of AAPH (600 μM) dissolved in Hank’s Balanced Salt Solution. The fluorescence intensity (emission at 538 nm, excitation at 485 nm) was recorded every 5 min for 1 h at 37 °C in a Fluoroskan Ascent FL plate-reader (Thermo Lab systems, Franklin, MA, USA). The CAA units were calculated as: CAA unit = 100 − (∫SA/∫CA) × 100. ∫SA refers to the area under curve of fluorescence intensity (AUC) of sample groups, while ∫CA refers to the AUC of control group. Five different molar ratios (1:10, 1:5, 1:1, 5:1, 10:1) of the phytochemical combinations [the combination of lutein and quercetin (LUT-Q); the combination of lutein and luteolin (LUT-L); the combination of lycopene and quercetin (LYP-Q); the combination of lycopene and luteolin (LYP-L)] were used to investigate the antioxidant synergistic/antagonistic effect.

### 2.5. Enzyme Activities of SOD, GSH-Px, and CAT

HUVEC, Caco-2, and L-02 cells (5 × 10^5^ cells per dish) were placed into culture dish 6 cm in diameter. HUVEC and L-02 reached 80–90% confluence and Caco-2 reached 100% confluence before treatments. After pre-treated with or without different concentrations of quercetin, luteolin, lycopene, lutein, or their combinations [M1 = lycopene:luteolin 1:5 (1.3 μM:6.7 μM), M2 = lycopene:quercetin 1:5 (1.3 μM:6.7 μM), M3 = lutein:luteolin 1:5 (1.3 μM:6.7 μM); M4 = lutein:luteolin 5:1 (6.7 μM:1.3 μM); M5 = lutein:quercetin 1:1 (4 μM:4 μM); M6 = lutein:quercetin 5:1 (6.7 μM:1.3 μM)] for 12 h, cells were incubated with H_2_O_2_ for another 1 h to induce oxidative stress. Superoxide dismutase (SOD), glutathione peroxidase (GSH-Px), and catalase (CAT) enzymatic activities were determined using colorimetric kits (Beyotime Biotech, Shanghai, China) according to the manufacturer’s protocols.

### 2.6. Measurement of Lycopene and Lutein Uptake in HUVEC, Caco-2, and L-02 Cells

HUVEC, Caco-2, and L-02 cells (5 × 10^5^ cells per dish) were placed into a culture plate 10 cm in diameter and reached 100% confluence before treatments. Cells were treated with 8 mL of Lycopene or lutein at 1.6 μM, and the combinations [M1 = lycopene:luteolin 1:5 (1.6 μM:8 μM), M2 = lycopene:quercetin 1:5 (1.6 μM:8 μM), M3 = lutein:luteolin 1:5 (1.6 μM:8 μM); M4 = lutein:luteolin 5:1 (1.6 μM:0.32 μM); M5 = lutein:quercetin 1:1 (1.6 μM:1.6 μM); M6= lutein:quercetin 5:1 (1.6 μM:0.32 μM)] for 12 h. Carotenoids extraction from cell lysates were followed the protocol described by Biehler et al. [17] with some modification. Cells were lysed with 1% Triton X-100 containing 1% protease inhibitor as lysis solution. Then the carotenoids in cells were extracted using hexane:ethanol:acetone (500 μL, 2:1:1, *v*/*v*/*v*) three times and dried by nitrogen gas and redissolved in 200 μL acetone for determination of cell content by High Performance Liquid Chromatograph (HPLC) analysis.

The cell contents of carotenoids were measured with an Agilent 1260 series HPLC system equipped with an autosampler (G1329B, serial number: DEAAC20184), a degasser (G1322A, serial number JPAAL87189), a quaternary pump (G1311C, serial number: DEAB815159), a diode array detector (DAD) (G1315B, serial number: DE40522724), and ChemStation software and separated on a Inertsil ODS-3 column (5 µm, 250 × 4.6 mm, GL Sciences Inc., Japan). The HPLC method for identification of carotenoids was conducted according to Reboul et al. [11] with slightly modification. The carotenoids were subjected to isocratic elution with the mobile phase consisting of 30% dichloromethane, 35% acetonitrile, and 35% methanol for 20 min. The injection volume was 20 µL, and the flow rate was 0.5 mL/min. Peaks were monitored at 450 nm and were tentatively identified by matching the retention time (RT) and UV absorption spectra with lycopene and lutein standards. Quantification of the cell content was achieved using standard curves generated from the peak area of lycopene (0.5–15 µM; y = 94.477x − 15.747, R^2^ = 0.999) and lutein (0.3–5 µM; y = 118.45x − 36.043, R^2^ = 0.99). Cell uptake (%) = the detected cell content/the initial added content.

### 2.7. Western Blot Analysis

Cell extracts (about 25 μg) were separated by 10% sodium dodecyl sulphate-polyacrylamide gel electrophoresis (SDS-PAGE) and transferred to polyvinylidene fluoride (PVDF) membranes (Roche Diagnostics GmbH, Mannheim, Germany). The membrane was blocked in 5% skim milk for 2 h and incubated with primary antibodies overnight at 4 °C. PVDF membranes were washed with TBST and incubated with secondary antibodies for 2 h at room temperature and visualized by ECL reagent and detected using enhanced chemiluminescence detection system (Image Lab™ Touch Software, BIO-RAD, Hercules, CA, USA). An image analyzer (ImageLab, BIO-RAD, USA) was used to determine the band intensity. Relative expression of proteins was normalized to β-actin.

### 2.8. Statistical Analysis

Statistical analysis was performed with IBM SPSS Statistics 20 software. Data is presented as mean ± standard deviation (SD) (*n* = 3). Significant differences between and within multiple groups were examined using one-way ANOVA with Duncan’s test and *p* < 0.05 was considered as statistically significant.

## 3. Results

### 3.1. Effects of Flavonoids and Carotenoids on the Oxidative-Induced Cell Damage

**Figure 2:**
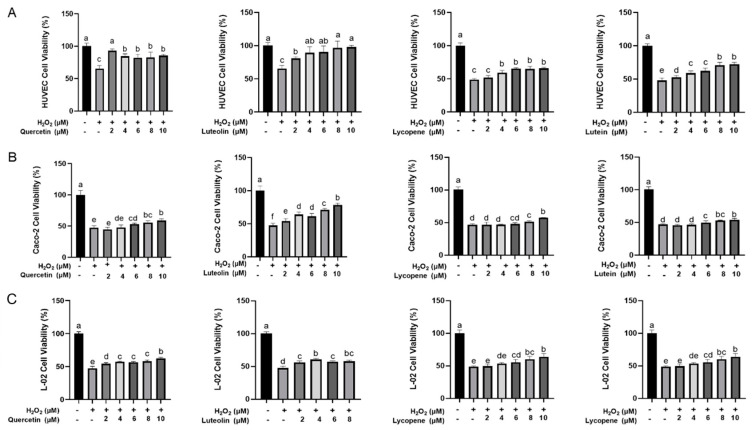
The cytoprotective effects of flavonoids (quercetin, luteolin) and carotenoids (lycopene, lutein) on the H_2_O_2_-induced oxidative damage in (**A**) HUVEC, (**B**) Caco-2, and (**C**) L-02 cells. Cells were incubated with a series of concentration of flavonoids or carotenoids for 12 h and then treated with H_2_O_2_ for 1 h. Results were presented as mean values ± SD (*n* = 4). Different letters indicate significant differences (*p* < 0.05) in Duncan’s test.

The cell viability was evaluated to assess the preventive effects of flavonoids (quercetin, luteolin) and carotenoids (lycopene, lutein) on oxidative stress in a series of concentrations from 0 to 10 μM. Luteolin mitigated H_2_O_2_ induced damage significantly at 2 μM (*p* < 0.05) in HUVEC (Figure 2A), Caco-2 (Figure 2B), and L-02 cells (Figure 2C). Quercetin ameliorated the oxidative damage caused by H_2_O_2_ at 2–10 μM in HUVEC and L-02 cells, and at 4–10 μM in Caco-2 cells. Lycopene and lutein protected cells from oxidative damage at relatively higher concentration: lycopene exerted protective effects at 4–10 µM in HUVEC (Figure 2A), 6–10 µM in Caco-2 and L-02 cells (Figure 2B,C), and lutein reversed cell damage at 2–10 µM in HUVEC (Figure 2A), 6–8 µM in Caco-2 (Figure 2B) and 2–8 µM in L-02 cells (Figure 2C). These four phytochemicals exhibited the strongest preventive effects on H_2_O_2_-induced oxidative damage in HUVEC cells. Since the cell viability was restored by up to 30% in HUVEC cells, around 12% in Caco-2 cells, and below 15% in L-02 cells. Considering that luteolin did not show cell toxicity in L-02 cells at 8 μM (showed cytotoxicity at 10 μM, Figure S3), 8 μM was selected as the total concentration for the individual or combined phytochemicals to assess the antioxidant interactions in three cell lines.

### 3.2. Interactions on Cellular Antioxidant Activity

CAA is an efficient model in evaluating the ability of an antioxidant to scavenge cellular peroxyl radicals generated from AAPH [18]. The CAA value of individual flavonoid and carotenoid in different cell types were presented in Figure S5. Four combinations in five ratios (1:10, 1:5, 1:1, 5:1 and 10:1) were applied to determine their CAA values (Table 1). These combinations showed almost synergistic effects in HUVEC cells, except for that the LUT-Q combinations at 5:1 exhibited a antagonistic effect. In general, the combination of lycopene with two flavonoids exhibited stronger synergistic effects than those combinations containing lutein. In Caco-2 cells, 65% combinations showed synergistic effects, especially those combinations contained quercetin. The LYP-Q combinations showed stronger synergistic effects, as all mixtures in different ratios showed synergistic effects. While the LYP-L combinations mainly showed antagonism. In L-02 cells, half of the combinations showed significant antagonistic effects, while a quarter of the combinations exhibited synergistic effects. Especially, synergy appeared at the fixed ratio (1:5) of the combinations in L-02 cells, except the LUT-Q combinations. Among the antagonistic group, the combination of lutein with two flavonoids exhibited stronger antagonistic effects than the combination of lycopene with flavonoids. A total concentration of six relatively strong synergistic groups (M1: LYP-L = 1:5; M2: LYP-Q = 1:5; M3: LUT-L = 1:5) or antagonistic groups (M4: LUT-L = 5:1; M5: LUT-Q = 1:1; M6: LUT-Q = 5:1) were chosen for further confirmation of antioxidant interactions, which were bolded in Table 1.

### 3.3. Interactions of Phytochemicals on SOD, GSH-Px, and CAT Activity

**Figure 3:**
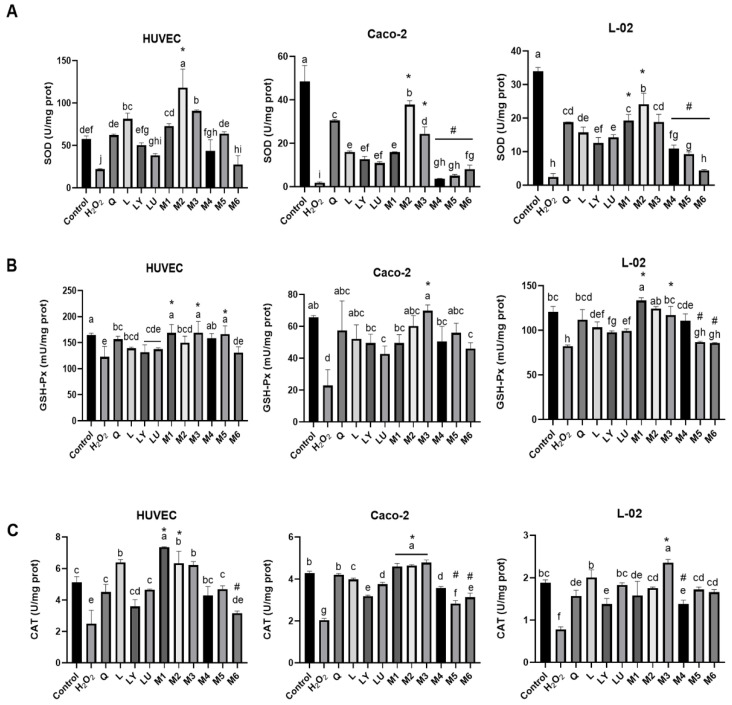
Effects of phytochemical combinations on (**A**) SOD, (**B**) GSH-Px, and (**C**) CAT activities in HUVEC, Caco-2, and L-02 cells. Cells were incubated with individual flavonoid or carotenoid, or their combinations for 12 h and then treated with H_2_O_2_ for 1 h. Q: quercetin; L: luteolin; LY: lycopene; LU: lutein; M1: LYP:L = 1:5; M2: LYP:Q = 1:5; M3: LUT:L = 1:5; M4: LUT:L = 5:1; M5: LUT:Q = 1:1; M6: LUT:Q = 5:1. Results were presented as mean values ± SD (*n* = 3). Different letters indicate significant differences (*p* < 0.05) in Duncan’s test. “*” indicates the synergistic effects, representing the enzyme activity of phytochemical combinations is stronger than individual ones (*p* < 0.05), while “#” indicates the antagonistic effects, representing the enzyme activity of phytochemical combinations is weaker than individual ones (*p* < 0.05). The combinations showed stronger activity than both individuals (*p* < 0.05) were considered as synergistic effects, the combinations showed weaker activity than both individuals (*p* < 0.05) were considered as antagonistic effects, and the combinations showed no higher or weaker activity than both individuals (*p* > 0.05) were considered as additive effects.

Flavonoids and carotenoids significantly restored the antioxidant enzyme activity impaired by H_2_O_2_ (Figure 3). All four phytochemicals boosted SOD activity (Figure 3A). Quercetin exhibited the strongest effects in Caco-2 and L-02 cells. Luteolin treatment showed the strongest SOD activity in HUVEC, increased 59 U/mg prot than H_2_O_2_ treatment. Four phytochemicals significantly facilitated GSH-Px activity in Caco-2 and L-02 cells but did not significantly promote the GSH-Px activity in HUVEC cells (Figure 3B). Luteolin showed the strongest activity in inducing CAT activity among four phytochemicals (Figure 3C).

Compared to the individual ones, flavonoid-carotenoid combinations M2 and M3 showed the strongest synergy, as they showed stronger activity on the antioxidant enzyme activities than their individual flavonoid or carotenoid (*p* < 0.05). While M6 showed the strongest antagonistic effects, as it showed weaker activity than both lutein and quercetin (*p* < 0.05). As shown in Figure 3A, M2 and M3 showed the strongest synergy, while M6 showed the strongest antagonistic effects in promoting SOD activity. GSH-Px activities were promoted in M3, while decreased in M5 and M6 compared to the individual treatments (Figure 3B). Moreover, M2 showed the strongest synergy, while M4 and M6 possessed the strongest antagonistic effects on facilitating CAT activity in three cell lines (Figure 3C). These results may suggest the strongest synergy occur when flavonoids in the majority of combination (M2, M3). Collectively, both individual and combined phytochemicals showed the strongest effects in promoting the antioxidant enzymes activity in HUVEC cells, suggesting the possible role of flavonoids and carotenoids in vascular protection through enhancing the antioxidant enzymes activity.

### 3.4. Flavonoids Affect the Uptake of Carotenoids

**Figure 4:**
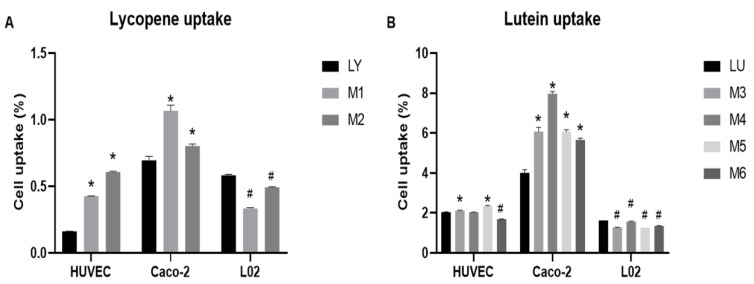
Effects of flavonoids on cell uptake of (**A**) lycopene and (**B**) lutein in HUVEC, Caco-2, and L-02 cells. Cells were incubated with individual carotenoid or flavonoid-carotenoid combinations for 12 h. “*” indicates a higher uptake rate of mix group than single group (*p* < 0.05), while “#” indicates a reduced uptake rate of mix group than single group (*p* < 0.05). M1: LYP:L = 1:5; M2: LYP:Q = 1:5; M3: LUT:L = 1:5; M4: LUT:L = 5:1; M5: LUT:Q = 1:1; M6: LUT:Q = 5:1.

As shown in Figure 4 and Figure S6, the uptake of lycopene is about 0.16–0.7% in different cell lines after being treated with lycopene for 12 h, up to 0.7% were detected in Caco-2 cells, and 0.58% were detected in L-02 cells. Lutein showed a higher uptake rate than lycopene, from 1.2% to 4% in different cell lines, and the highest uptake rate was also detected in Caco-2 cells. These results were in line with the previous research that the absorption of lycopene was lower than lutein in Caco-2 cells [19]. The uptake of lycopene was increased in M1 and M2, which indicated that quercetin and luteolin significantly increased the uptake of lycopene in HUVEC cells (Figure 4A). The lycopene uptake increased by 271% in M2 than the individual lycopene uptake. The lutein uptake was increased in M3 and M5. While the lutein uptake decreased by approximately 17% in M6 compared to the individual lutein uptake (Figure 4B). These results may imply a positive correlation between antioxidant interactions and the effects of flavonoids on the uptake of carotenoids, as M1–M5 showed synergistic effects while M6 exhibited strong antagonistic effects on CAA and the antioxidant enzyme activities in HUVEC. In Caco-2 cells, the uptake of lycopene also increased in M1 and M2 (Figure 4A). lycopene uptake was increased around 53% after treated with M1. The lutein uptake was significantly increased in selected combinations (M3–M6) (Figure 4B). Especially, lutein uptake was promoted by 98% after treated with M4. In L-02 cells, an impaired lycopene uptake in M1 and M2 were detected. Lutein uptake was also significantly reduced in M3–M6.

### 3.5. The Presence of Flavonoids Influenced the Expression of Carotenoid Transporters

**Figure 5:**
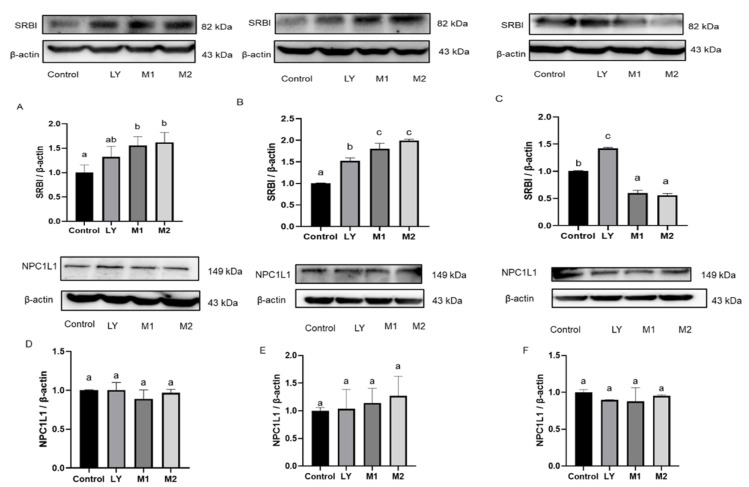
Effects of lycopene and flavonoids combinations on the expression of scavenger receptor class B type I (SR-BI) in (**A**) HUVEC, (**B**) Caco-2, and (**C**) L-02 cells, and Niemann-Pick C1-like 1(NPC1L1) in (**D**) HUVEC, (**E**) Caco-2, and (**F**) L-02 cells. LY: lycopene, M1: LYP:L = 1:5; M2: LYP:Q = 1:5. After incubated by these phytochemicals for 12 h, the expressions of SR-BI and NPC1L1 were detected. The band shows the immunoblot of one experiment representing SR-BI or NPC1L1 expression. β-actin was used as an internal reference. Values are expressed as the mean ± SD (*n* = 3). Values with different letters above the column represent a significant difference (*p* < 0.05).

**Figure 6:**
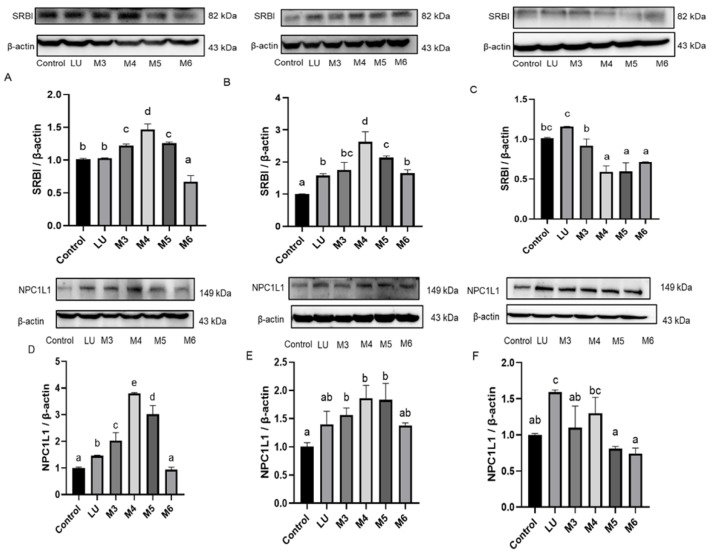
Effects of lutein and flavonoids combinations on the expression of SR-BI in (**A**) HUVEC, (**B**) Caco-2, and (**C**) L-02 cells, and NPC1L1 in (**D**) HUVEC, (**E**) Caco-2, and (**F**) L-02 cells. LU: lutein, M3: LUT:L = 1:5; M4: LUT:L = 5:1; M5: LUT:Q = 1:1; M6: LUT:Q = 5:1. After incubated by these phytochemicals for 12 h, the expressions of SR-BI and NPC1L1 were detected. The band shows the immunoblot of one experiment representing SR-BI or NPC1L1 expression. β-actin was used as an internal reference. Values are expressed as the mean ± SD (*n* = 3). Values with different letters above the column represent a significant difference (*p* < 0.05).

The uptake of carotenoids was reported to be partly facilitated by active transporters, such as SR-BI and NPC1L1 [16]. In HUVEC, SR-BI were increased by lycopene in a non-significant way (Figure 5A). Treated with quercetin or luteolin alone could not induce the change of SR-BI (Figure S7). M1 and M2 significantly promoted the expression of SR-BI than cell lysates treated with lycopene. It may suggest that when co-existing with flavonoids, SR-BI was activated and therefore promoting the lycopene uptake in HUVEC. Lutein induced the expression of SR-BI significantly. M3, M4, and M5 increased SR-BI expression, while M6 significantly inhibited the SR-BI expression compared to the treatment of lutein alone (Figure 6A). The expression of SR-BI exhibited the same trends as the uptake of lycopene and lutein. In Caco-2 cells, M1 and M2 significantly promoted the expression of SR-BI than the individual lycopene group (Figure 5B). M4 and M5 also significantly promoted the expression of SR-BI than the individual lutein group (Figure 6B). In L-02 cells, M1 and M2 significantly inhibited the expression of SR-BI than the treatment of lycopene alone (Figure 5C). Similarly, M3–M6 significantly reduced the expression of SR-BI compared to lutein group (Figure 6C).

NPC1L1 is a versatile transporter involved in the transport of lutein. Individual treatment of quercetin or luteolin could not induce the change of NPC1L1 (Figure S7). NPC1L1 expression could not be increased by lycopene and lycopene mixtures (M1, M2, Figure 5D–F). In HUVEC cells, lutein induced NPC1L1 expression, and lutein combination M3, M4, and M5 significantly induced the NPC1L1 expression compared to the single treatment of lutein, while M6 reduced the NPC1L1 expression (Figure 6D). In Caco-2 cells, lutein could not significantly induced NPC1L1 expression, and lutein combination M3, M4, M5 increased the NPC1L1 expression compared to the treatment of lutein alone. In L-02 cells, lutein mixtures M3, M5, and M6 decreased the expression of NPC1L1 (Figure 6F). Apparently, NPC1L1 was not involved in the transport of lycopene, which was in line with the previous research [20]. The effects of flavonoids on lutein uptake were in accordance with the effects of flavonoid-carotenoid combinations on the expression of NPC1L1. Therefore, the presence of flavonoids may enhance or impair carotenoids uptake through affecting the expression of SR-BI and NPC1L1 transporters.

## 4. Discussion

In the current study, flavonoids and carotenoids exhibited antioxidant interactions through the regulation of ROS scavenging ability and antioxidant enzyme activities (SOD, CAT, GSH-Px). Further, the effects of flavonoids on the uptake of carotenoids were demonstrated in HUVEC, Caco-2, and L-02 cells. The presence of flavonoids enhanced or decreased the uptake of carotenoids, which may be via the alteration of carotenoid transporters SR-BI and NPC1L1.

Both the ratios and cell types influenced the antioxidant interactions of phytochemicals as indicated by ROS scavenging and antioxidant enzyme activities. In general, the antioxidant activity of the combinations was stronger when flavonoids were in the majority. Flavonoids were reported to react with the oxidized carotenoids to regenerate them. Baicalin combined with β-carotene at molecular ratio of 1:1 could inhibit the lipoxidation better than the individual baicalin as it could regenerate β-carotene with a second-order rate constant of (5.6 ± 0.5) × 10^9^ L mol^−1^ s^−1^ in the methanol/chloroform binary solvent (1:9, *v*/*v*) [21]. Anthocyanins were also shown to improve carotenoid stability in comparative studies of purple tomatoes and related red tomatoes [22]. Therefore, it was possible that flavonoids could regenerate carotenoids when the flavonoids form the majority in the flavonoid-carotenoid mixture and resulted in synergistic effects.

The antioxidant interactions and the bioavailability interactions among phytochemicals were often measured independently, however, rare study investigated their correlation [23]. Several studies have revealed the role of flavonoids in the cell uptake of carotenoids without further determining their influence on the biological effects of carotenoids [11,24,25,26]. The absorption, distribution, and metabolism of carotenoids varied in different tissues. Therefore, the influence of flavonoids on the uptake of carotenoids and antioxidant activity may also be tissue- or cell-specific. In HUVEC cells, M1–M5 showed increased lycopene/lutein uptake than either lycopene or lutein alone, whereas M6 treatment resulted in impaired lutein uptake. Correspondently, M1–M5 demonstrated synergistic effects, while M6 exhibited antagonistic effects in ROS scavenging and elevation of antioxidant enzyme activities. Therefore, flavonoids may further influence antioxidant interactions through altering the uptake of carotenoids. In addition, both flavonoids and carotenoids exhibited the strongest antioxidant activity in HUVEC cells than those in Caco-2 or L-02 cells. The antioxidant synergistic combinations also enhanced the uptake of carotenoids, while the antagonistic combinations hampered the uptake of carotenoids. It may indicate that phytochemicals showed potent vascular protection, and HUVEC cell is a desirable model for assessing antioxidant interactions. In Caco-2 cells, the strong synergies were found in M2 and M3, while the antagonistic effects were found in M4 and M5. Simultaneously, both quercetin and luteolin enhanced the uptake of lycopene or lutein in all combinations. In L-02 cells, M2 showed synergistic effects on the enhancement of antioxidant enzyme activities while M5 and M6 showed the antagonistic effects. However, carotenoids uptake was reduced when co-treated with flavonoids in L-02 cells. Therefore, flavonoids appeared to affect the uptake of carotenoids differently in different cell lines. This may be due to different tissue distributions and metabolic activities of carotenoids. Lycopene was found to be distributed differently in tissues, such as liver, intestine, and prostate in rats [27]. Jeon et al. [28] also reported that lutein was detectable in all tissues and total lutein was most concentrated in the macular retina, adrenal gland, and liver. Carotenoids were stored in the liver carotenoids and were re-secreted into the circulation and distributes them to other tissues. However, the L-02 cell model often reflects merely the uptake of carotenoids, as the absorption, incorporating into chylomicron, phase Ⅱ metabolism, and accumulation of carotenoids often need cooperation of multiple cells in small intestine, portal veins, and liver. It is possible that representing the intestinal cells, Caco-2 is more efficient in the uptake of carotenoids, while as a model of liver cells, carotenoids may absorb into L-02 cells and transport to blood and distributed to different tissues. Therefore, the detected lycopene and lutein content is lower in L-02 cells than in Caco-2 cells. Flavonoids were considered to interfere with the absorption of carotenoids in several studies [11,12,24,25,26,29]. In the case of naringenin and lutein, competition between them for cellular uptake led to the reduced lutein uptake in vivo [11]. Conversely, naringenin were reported to enhance β-carotene uptake up to 1.3-fold in intestinal Caco-2 cells [30]. In general, flavonoids are demonstrated to have low bioavailability, owing to the poor absorption and fast metabolization and excretion. It is hard to quantify the uptake of flavonoids as they were transformed to diverse metabolites and undergo rapid excretion by efflux pumps such as multidrug resistance protein and P-glycoprotein [31]. Therefore, the effects of carotenoids on the cell uptake of flavonoids were not determined in this study.

The absorption of carotenoids into intestine is not just passive but also involves specific epithelial transporters, such as SR-BI, NPC1L1, and cluster determinant 36 (CD 36). In the current study, lutein might be transported into cells by SR-BI and NPC1L1, and SR-BI but not NPC1L1 was activated after being treated with lycopene, as the expression of SR-BI were increased. Flavonoid-carotenoid combinations changed the expression of SR-BI and NPC1L1 compared to the single treatment of carotenoid, which was in accordance with the effects of flavonoids on carotenoids uptake. It indicated a close relationship between the effects of flavonoids on the cell uptake of carotenoids and the regulation of carotenoid transporters. Flavonoids usually locate in the hydrophilic compartment of the lipid membrane due to its hydrophilicity. Once being incorporated into bilayer, the polarization area increased, promoting the membrane fluidization. As a result, the structure of lipid raft, a specific domain in the membrane mainly constitute by cholesterol and saturated sphingolipids, would be altered. Various transport proteins usually located in caveolae/lipid raft microdomains, such alteration may influence the diffusion of lipophilic compounds such as carotenoids through the cell membrane and further influenced their uptake into cells. Thus, the enhanced or decreased expression of transporters by flavonoid-carotenoid combinations may be related to the interaction of flavonoids with cell membrane lipid rafts. For instance, the presence of citrus flavonoids, such as hesperidin were reported to enhance the absorption of β-carotene through elevating the expression of SR-BI in Caco-2 cells [26]. Previously, we found that the antioxidant synergism among phenolic acids and β-carotene was possible due to the improved expression of carotenes cell membrane transporters CD36 and SR-BI by phenolic acid-carotene combinations [32]. In addition, flavonoids could also influence the uptake of carotenoids by changing the membrane fluidity or paracellular permeability. Flavonoids such as hesperetin and naringenin were reported to affects the paracellular permeability through transient reduction of the protein levels of tight junction proteins ZO-1 and claudin-1 [26]. In addition, the structure of the co-existed flavonoids, such as number of phenolic groups and metabolized form, may also influence the bioaccessibility of carotenoids [33,34]. It was reported that hesperidin, the glycosylated form of hesperetin, was more efficient in improving the β-carotene’s micellization and bioaccessibility than its aglycone form [35].

In summary, antioxidant interaction was found among flavonoid-carotenoid combinations in HUVEC, Caco-2, and L-02 cells, which varied with the change of combination ratios and cell types. Flavonoids affected the uptake of carotenoids by altering the expression of transporters SR-BI and NPC1L1, which may contribute to their antioxidant interactions. Our results provided new insights into the mechanisms responsible for antioxidant interactions among different phytochemicals. It may provide theoretical basis for nutritionally balanced diet containing both of flavonoids and carotenoids. Future work should focus on the relationship between antioxidant interactions and bioavailability among phytochemicals in vivo by animal models or clinical research.

## Figures and Tables

**Table 1:** Synergistic or antagonistic effects on cellular antioxidant activity (CAA) of phytochemical combinations in HUVEC, Caco-2, and L-02 cells.

Combination	HUVEC Cells				Caco-2 Cells			L-02 Cells		
	Ratio	CAA_Theo_ (%) ^a^	CAA_Exp_ (%) ^b^	Effect ^c^	CAA_Theo_ (%)	CAA_Exp_ (%)	Effect	CAA_Theo_ (%)	CAA_Exp_ (%)	Effect
LUT-Q	1:10	38.3 ± 2.71	43.1 ± 4.66	Synergy ^d^	31.7 ± 0.97	41.8 ± 3.49	Synergy	71.8 ± 4.91	36.9 ± 2.83	Antagonism^e^
	1:5	36.9 ± 2.60	37.1 ± 3.69	Additivity ^f^	31.3 ± 1.02	40.4 ± 1.48	Synergy	68.1 ± 1.24	26.7 ± 2.46	Antagonism
	**1:1**	**30.7 ± 2.12**	**36.3 ± 2.30**	**Synergy**	**29.6 ± 1.23**	**24.7 ± 2.60**	**Antagonism**	**69.7 ± 2.89**	**68.9 ± 4.15**	**Additivity**
	**5:1**	**24.5 ± 1.64**	**19.0 ± 1.30**	**Antagonism**	**28.0 ± 1.44**	**36.6 ± 3.88**	**Synergy**	**71.4 ± 4.54**	**59.1 ± 3.15**	**Antagonism**
	10:1	23 ± 1.53	27.9 ± 1.97	Synergy	27.6 ± 1.49	34.1 ± 2.99	Synergy	71.8 ± 4.91	41.1 ± 3.31	Antagonism
LUT-L	1:10	42.2 ± 3.48	53.7 ± 4.13	Synergy	42.3 ± 2.21	37.0 ± 1.75	Antagonism	75.8 ± 3.94	68.9 ± 1.30	Antagonism
	**1:5**	**40.4 ± 3.30**	**54.8 ± 3.65**	**Synergy**	**41.1 ± 2.15**	**50.7 ± 1.62**	**Synergy**	**68.8 ± 1.06**	**82.5 ± 6.61**	**Synergy**
	1:1	32.8 ± 2.54	42.6 ± 3.80	Synergy	35.5 ± 1.91	34.8 ± 3.82	Additivity	71.9 ± 2.35	73.1 ± 50	Additivity
	**5:1**	**25.2 ± 1.78**	**44.3 ± 4.66**	**Synergy**	**29.9 ± 1.67**	**18.2 ± 0.73**	**Antagonism**	**75.0 ± 3.64**	**63.7 ± 4.86**	**Antagonism**
	10:1	23.4 ± 1.61	39.8 ± 3.46	Synergy	28.7 ± 1.61	34.8 ± 3.46	Synergy	75.78 ± 3.94	33.9 ± 2.15	Antagonism
LYP-Q	1:10	38.6 ± 2.73	46.4 ± 3.65	Synergy	31.0 ± 0.91	41.2 ± 2.63	Synergy	71.6 ± 5.27	80.5 ± 3.46	Synergy
	**1:5**	**37.4 ± 2.64**	**55.3 ± 2.24**	**Synergy**	**30.0 ± 0.92**	**42.0 ± 3.82**	**Synergy**	**66.8 ± 4.47**	**78.6 ± 2.96**	**Synergy**
	1:1	32.2 ± 2.25	51.8 ± 5.70	Synergy	25.8 ± 0.93	35.4 ± 2.55	Synergy	68.9 ± 4.83	69.4 ± 4.46	Additivity
	5:1	27.0 ± 1.85	54.5 ± 5.89	Synergy	21.5 ± 0.94	41.1 ± 3.76	Synergy	71.1 ± 5.18	56.7 ± 2.35	Antagonism
	10:1	25.8 ± 1.76	42.5 ± 7.73	Synergy	20.6 ± 0.94	31.1 ± 3.30	Synergy	71.6 ± 5.27	66.7 ± 3.82	Additivity
LYP-L	1:10	42.4 ± 3.50	47.0 ± 2.59	Synergy	41.6 ± 2.15	38.2 ± 1.71	Antagonism	75.6 ± 4.28	77.9 ± 1.79	Additivity
	**1:5**	**40.9 ± 3.34**	**44.7 ± 7.20**	**Synergy**	**39.8 ± 2.05**	**25.3 ± 1.82**	**Antagonism**	**67.5 ± 4.16**	**84.4 ± 5.54**	**Synergy**
	1:1	34.3 ± 2.67	52.5 ± 2.58	Synergy	31.6 ± 1.61	26.3 ± 1.68	Antagonism	71.1 ± 4.21	71.1 ± 4.65	Additivity
	5:1	27.7 ± 1.99	52.1 ± 9.59	Synergy	23.5 ± 1.16	35.7 ± 2.46	Synergy	74.8 ± 4.26	59.2 ± 3.62	Antagonism
	10:1	26.2 ± 1.84	49.3 ± 9.78	Synergy	21.6 ± 1.06	27.6 ± 2.51	Synergy	75.6 ± 4.28	52.7 ± 2.62	Antagonism

^a^ LUT-Q: the combination of lutein and quercetin; LUT-L: the combination of lutein and luteolin; LYP-Q: the combination of lycopene and quercetin; LYP-L: the combination of lycopene and luteolin. ^b^ CAA_Theo_: The theoretical CAA units of phytochemical combinations. For example, the LUT-Q combined at 1:10: CAA_Theo_ = 1/11 × CAA_LUT_ + 10/11 CAA_Q_. CAA_LUT:_ The CAA units of LUT at 8 μM. CAA_Q_: The CAA units of Q at 8 μM. CAA unit = 100 − (∫SA/∫CA) × 100. ^c^ CAA_exp_: The experimental CAA units of phytochemical combinations. ^d^ Synergy: CAA_exp_ > CAA_Theo_ (*p* < 0.05); ^e^ Antagonism: CAA_exp_ < CAA_Theo_ (*p* < 0.05); ^f^ Additivity: CAA_exp_ = CAA_Theo_ (*p* < 0.05).

## Supplementary Materials

The following are available online at https://www.mdpi.com/article/10.3390/foods10123096/s1, Figure S1. Cytotoxicity of selected phytochemicals in a series of concentration for 12 h in HUVEC cells measured by CCK8 assay. Results were presented as mean values ± SD (*n* = 4), Figure S2. Cytotoxicity of selected phytochemicals in a series of concentrations for 12 h in Caco-2 cells measured by CCK8 assay. Results were presented as mean values ± SD (*n* = 4), Figure S3. Cytotoxicity of selected phytochemicals in a series of concentrations for 12 h in L-02 cells measured by CCK8 assay. Results were presented as mean values ± SD (*n* = 4). “*” indicates a significant decrease of cell viability by phytochemicals, indicating a significant cytotoxicity (*p* < 0.05), Figure S4. Cytotoxicity of H2O2 in a series of concentrations for 1 h in A. HUVEC, B. Caco-2, and C. L-02 cells measured by CCK8 assay. Results were presented as mean values ± SD (*n* = 4). “*” indicates a significant decrease of cell viability by phytochemicals, indicating a significant cytotoxicity (*p* < 0.05), Figure S5. Cell antioxidant activity of quercetin (Q), luteolin (L), lycopene (LY), lutein (LU) in HUVEC, Caco-2, and L-02 cells. Results were presented as mean values ± SD (*n* = 3). (*p* < 0.05). Different letter indicates a significant difference (*p* < 0.05) in Duncan’s test, Figure S6. The HPLC analysis of the cell uptake of carotenoids influenced by flavonoids. A. The HPLC profiles of lycopene and lutein standards at 5 μM. The UV-VIS absorbance of lycopene and lutein were detected at 470 nm and 450 nm, respectively. The uptake of lycopene in B. HUVEC, C. Caco-2, and D. L-02 cells. The uptake of lutein in E. HUVEC, F. Caco-2, and G L-02 cells, Figure S7. The effects of quercetin and luteolin on the expression of SR-BI in A. HUVEC, B. Caco-2, and C. L-02 cells, and NPC1L1 in D. HUVEC, E. Caco-2, and F. L-02 cells. Q: quercetin; L: luteolin. After incubated for 12 h, the expressions of SR-BI and NPC1L1 were detected. The band shows the immunoblot of one experiment representing SR-BI or NPC1L1 expression. β-actin was used as an internal reference. Values are expressed as the mean ± SD (*n* = 3).
