# Supplementary material for: Interaction between Flavonoids and Carotenoids on Ameliorating Oxidative Stress and Cellular Uptake in Different Cells
Source: Foods. 2021 Dec 14;10(12):3096. doi: 10.3390/foods10123096 (PMC8701200; doi:10.3390/foods10123096)
Supplement: Supplementary file 1 [file foods-10-03096-s001.zip › foods-1471138-supplementary.pdf]

Figure captions

Figure S1. Cytotoxicity of selected phytochemicals in a series of concentration for 12 h in HUVEC cells measured by CCK8 assay. Results were presented as mean values ± SD (n=4).

Figure S2. Cytotoxicity of selected phytochemicals in a series of concentrations for 12 h in Caco-2 cells measured by CCK8 assay. Results were presented as mean values ± SD (n=4).

Figure S3. Cytotoxicity of selected phytochemicals in a series of concentrations for 12 h in L-02 cells measured by CCK8 assay. Results were presented as mean values ± SD (n=4). "*" indicates a significant decrease of cell viability by phytochemicals, indicating a significant cytotoxicity ($p < 0.05$).

Figure S4. Cytotoxicity of H_2O_2 in a series of concentrations for 1 h in A. HUVEC, B. Caco-2, and C. L-02 cells measured by CCK8 assay. Results were presented as mean values ± SD (n=4). "*" indicates a significant decrease of cell viability by phytochemicals, indicating a significant cytotoxicity ($p < 0.05$).

Figure S5. Cell antioxidant activity of quercetin (Q), luteolin (L), lycopene (LY), lutein (LU) in HUVEC, Caco-2, and L-02 cells. Results were presented as mean values ± SD (n=3). ($p < 0.05$). Different letter indicates a significant difference (p< 0.05) in Duncan's test.

Figure S6. The HPLC analysis of the cell uptake of carotenoids influenced by flavonoids. **A.** The HPLC profiles of lycopene and lutein standards at 5 μM. The UV-VIS absorbance of lycopene and lutein were detected at 470 nm and 450 nm, respectively. The uptake of

lycopene in **B.** HUVEC, **C.** Caco-2, and **D.** L-02 cells. The uptake of lutein in **E.** HUVEC, **F.** Caco-2, and **G** L-02 cells.

Figure S7. The effects of quercetin and luteolin on the expression of SR-BI in **A**. HUVEC, **B**. Caco-2, and **C**. L-02 cells, and NPC1L1 in **D**. HUVEC, **E.** Caco-2, and **F.** L-02 cells. Q: quercetin; L: luteolin. After incubated for 12 h, the expressions of SR-BI and NPC1L1 were detected. The band shows the immunoblot of one experiment representing SR-BI or NPC1L1 expression. β-actin was used as an internal reference. Values are expressed as the mean ± SD (n = 3).

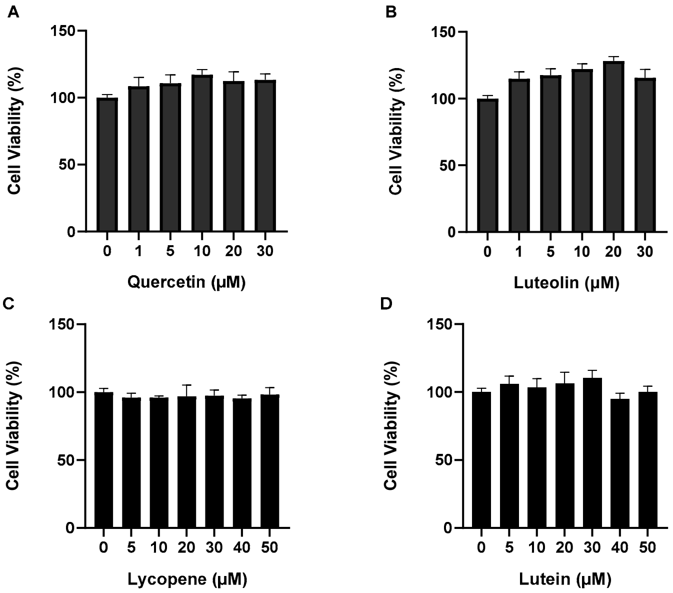

Figure S1. Cytotoxicity of selected phytochemicals in a series of concentration for 12 h in HUVEC cells measured by CCK8 assay. Results were presented as mean values ± SD (n=4).

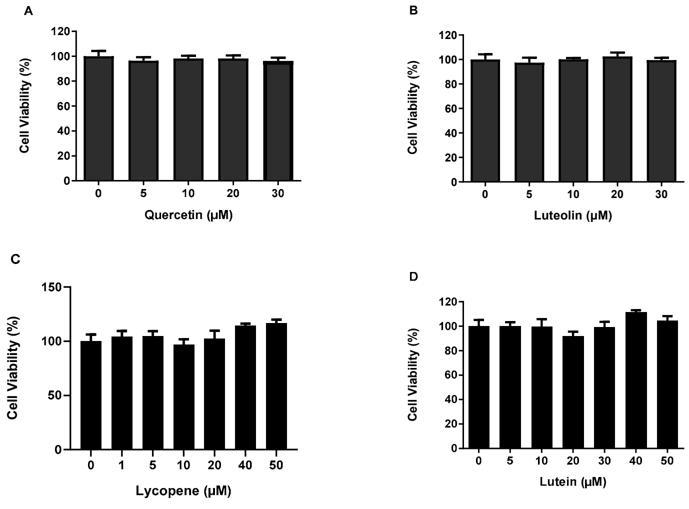

Figure S2. Cytotoxicity of selected phytochemicals in a series of concentrations for 12 h in Caco-2 cells measured by CCK8 assay. Results were presented as mean values ± SD (n=4).

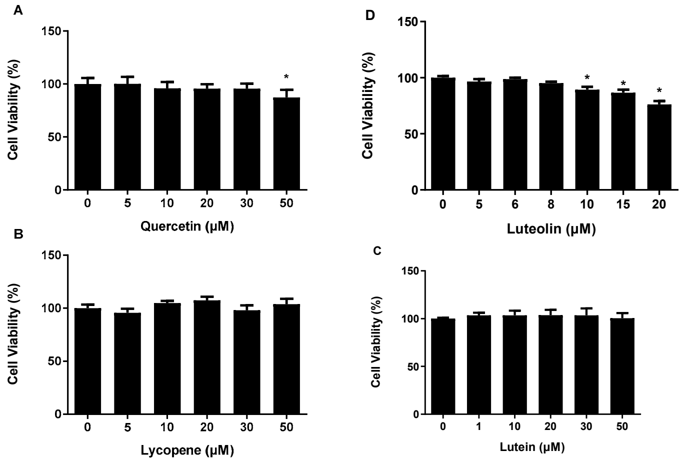

Figure S3. Cytotoxicity of selected phytochemicals in a series of concentrations for 12 h in L-02 cells measured by CCK8 assay. Results were presented as mean values ± SD (n=4). "*" indicates a significant decrease of cell viability by phytochemicals, indicating a significant cytotoxicity (p< 0.05).

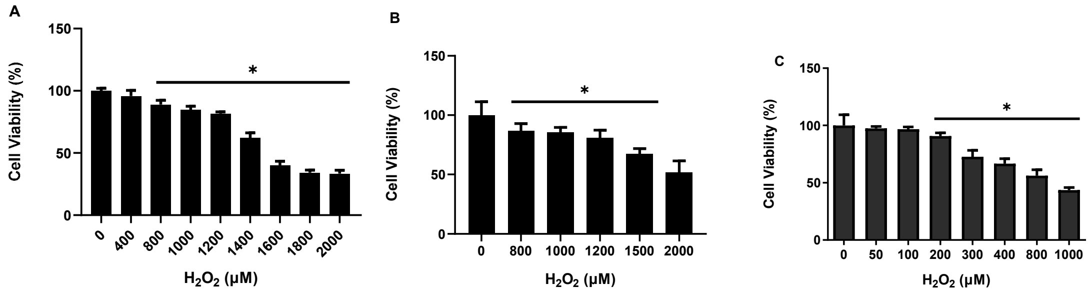

Figure S4. Cytotoxicity of H_2O_2 in a series of concentrations for 1 h in A. HUVEC, B. Caco-2, and C. L-02 cells measured by CCK8 assay. Results were presented as mean values ± SD (n=4). "*" indicates a significant decrease of cell viability by phytochemicals, indicating a significant cytotoxicity (p< 0.05).

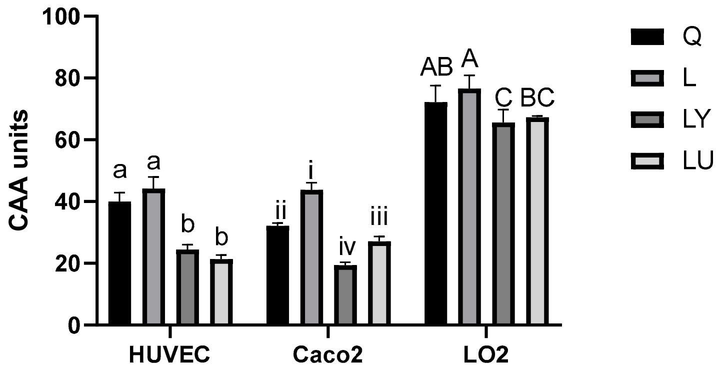

Figure S5. Cell antioxidant activity of quercetin (Q), luteolin (L), lycopene (LY), lutein (LU) in HUVEC, Caco-2, and L-02 cells. Results were presented as mean values ± SD (n=3). ($p < 0.05$). Different letter indicates a significant difference ($p < 0.05$) in Duncan's test.

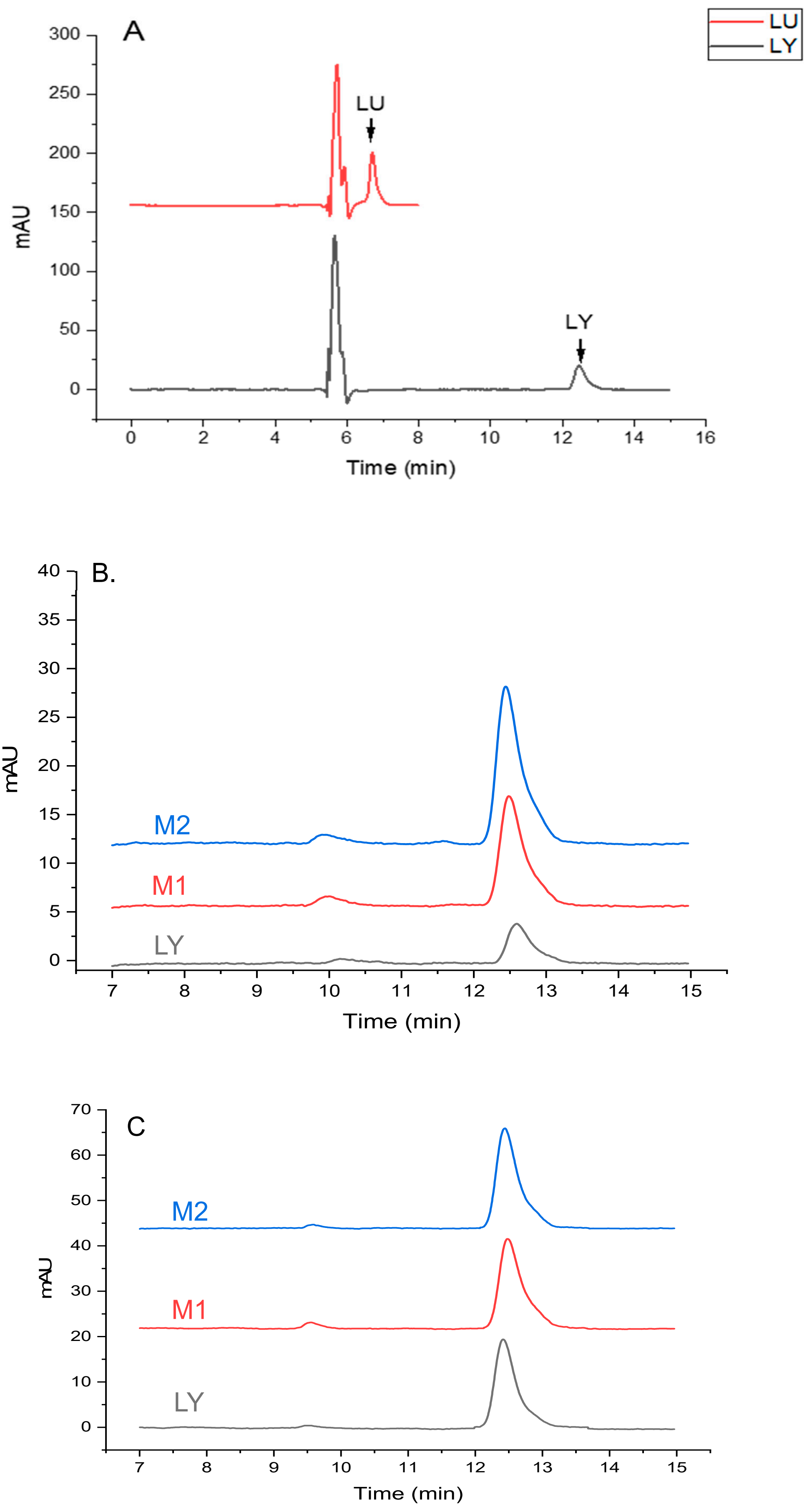

A
LU
LY
mAU
Time (min)
B.
M2
M1
LY
mAU
Time (min)
C
M2
M1
LY
mAU
Time (min)

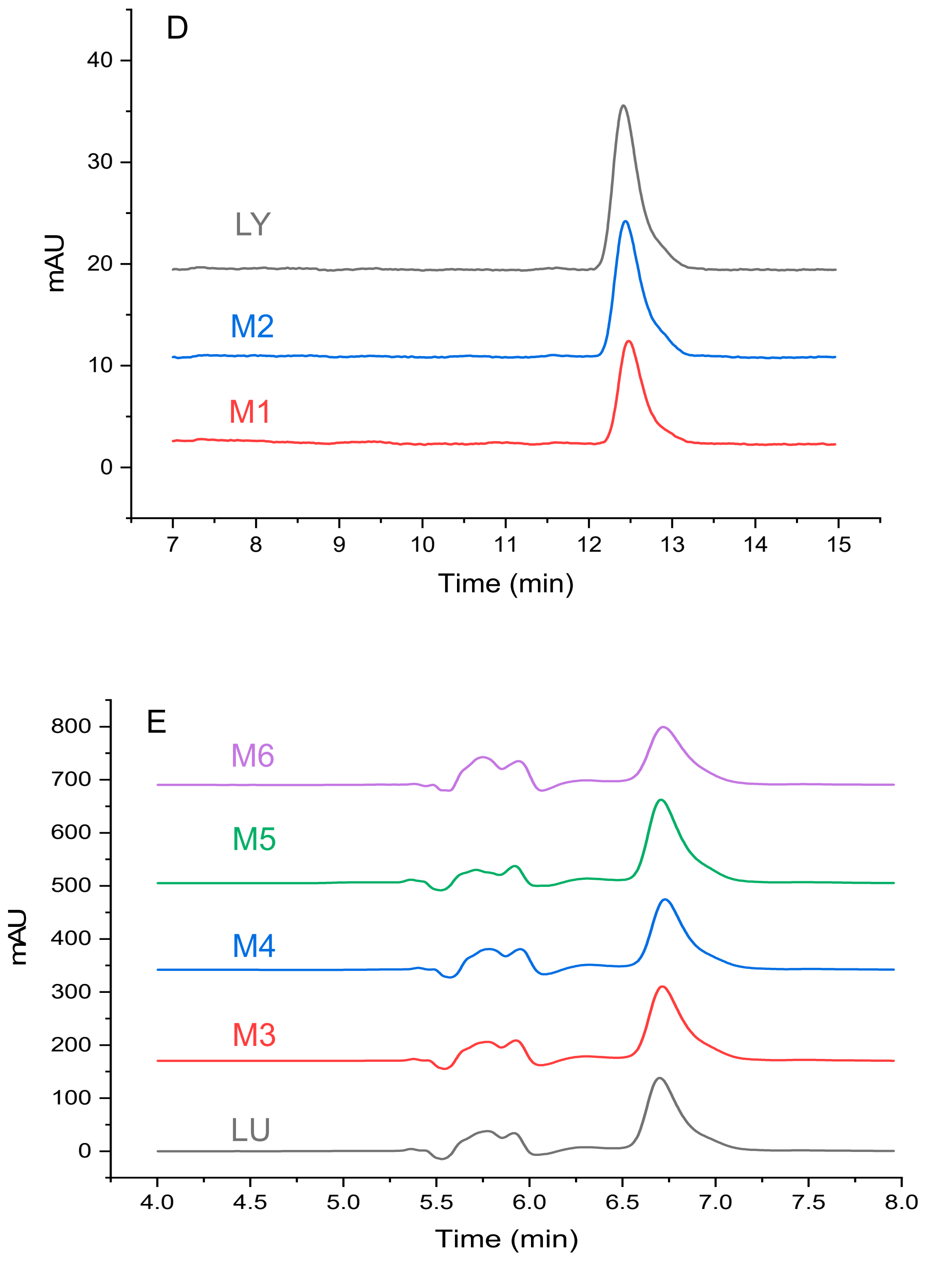
D
mAU
40
30
20
10
0
LY
M2
M1
7
8
9
10
11
12
13
14
15
Time (min)
E
mAU
800
700
600
500
400
300
200
100
0
M6
M5
M4
M3
LU
4.0
4.5
5.0
5.5
6.0
6.5
7.0
7.5
8.0
Time (min)

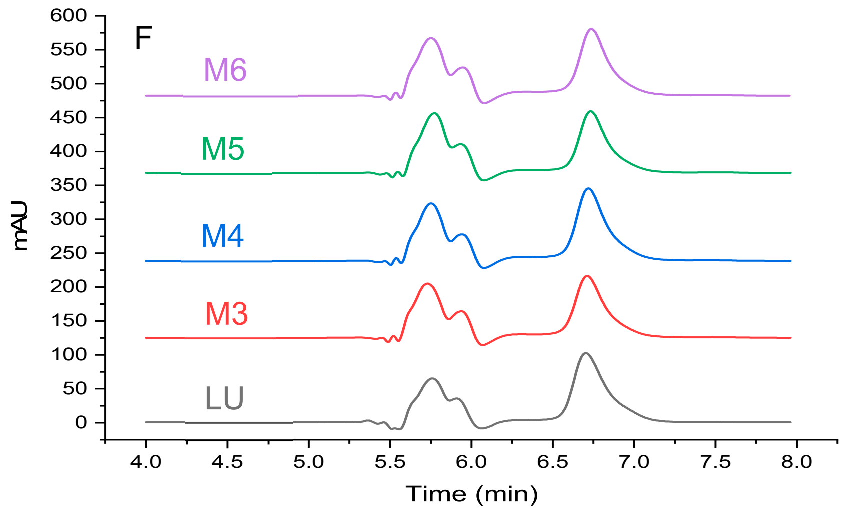

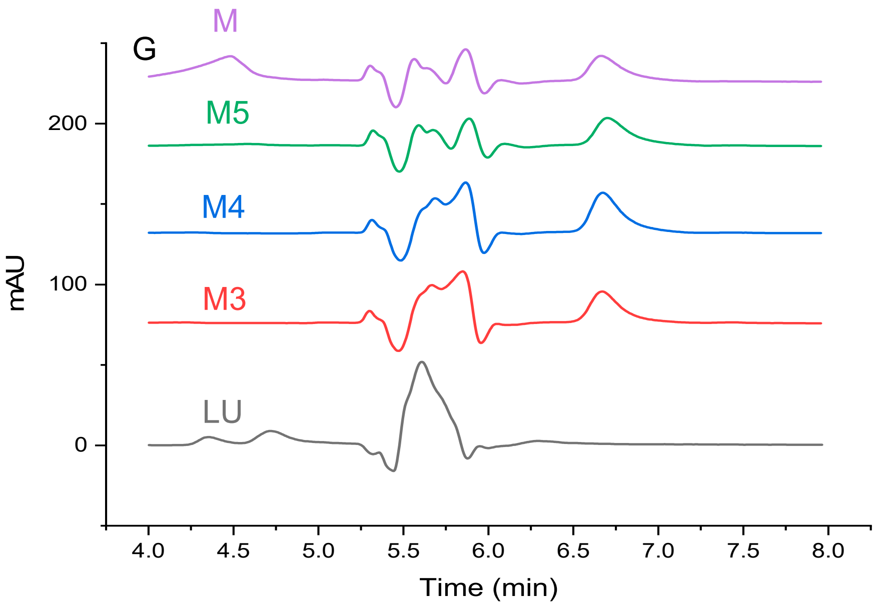

Figure S6. The HPLC analysis of the cell uptake of carotenoids influenced by flavonoids. **A.** The HPLC profiles of lycopene and lutein standards at 5 μM. The UV-VIS absorbance of lycopene and lutein were detected at 470 nm and 450 nm, respectively. The uptake of lycopene in **B.** HUVEC, **C.** Caco-2, and **D.** L-02 cells. The uptake of lutein in **E.** HUVEC, **F.** Caco-2, and **G** L-02 cells.

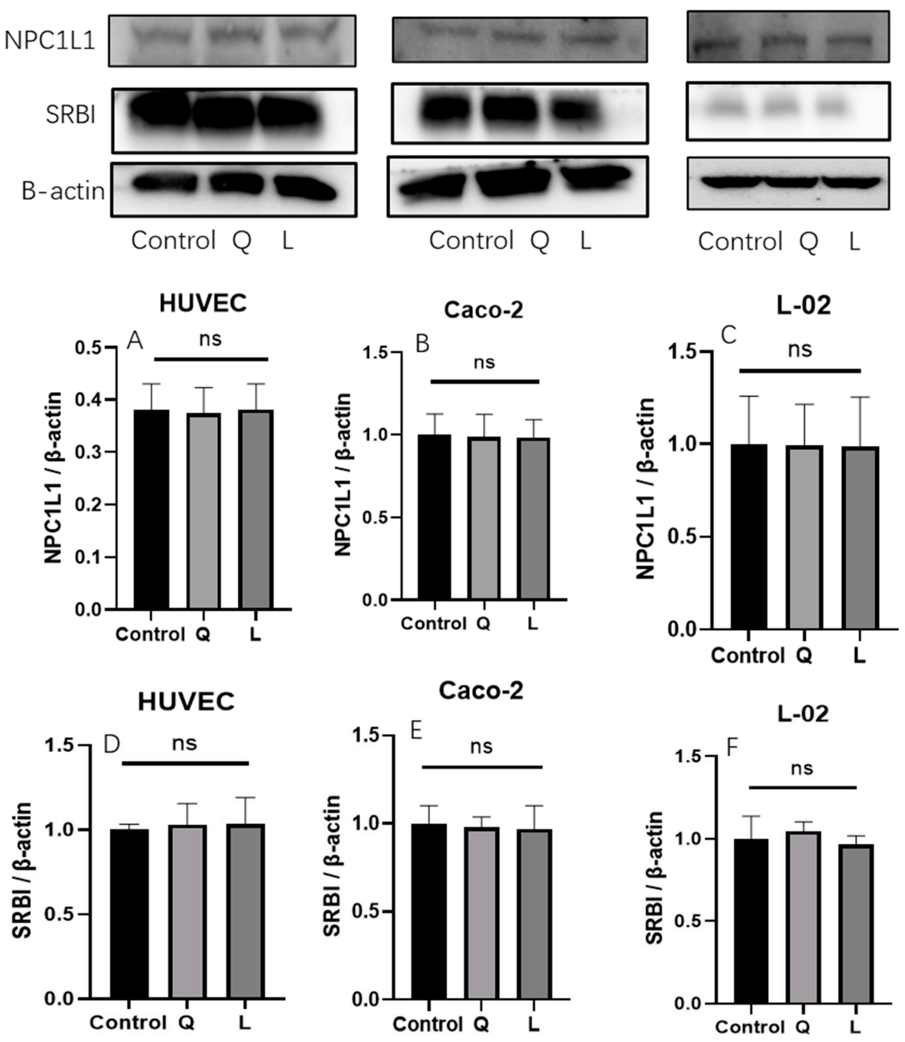

Figure S7. The effects of quercetin and luteolin on the expression of SR-BI in **A**. HUVEC, **B**. Caco-2, and **C**. L-02 cells, and NPC1L1 in **D**. HUVEC, **E.** Caco-2, and **F.** L-02 cells. Q: quercetin; L: luteolin. After incubated for 12 h, the expressions of SR-BI and NPC1L1 were detected. The band shows the immunoblot of one experiment representing SR-BI or NPC1L1 expression. β-actin was used as an internal reference. Values are expressed as the mean ± SD (n = 3).
